# Normal saline versus Ringer’s solution and critical-illness mortality in acute pancreatitis: a nationwide inpatient database study

**DOI:** 10.1186/s40560-024-00738-y

**Published:** 2024-07-15

**Authors:** Masayasu Horibe, Astuto Kayashima, Hiroyuki Ohbe, Fateh Bazerbachi, Yosuke Mizukami, Eisuke Iwasaki, Hiroki Matsui, Hideo Yasunaga, Takanori Kanai

**Affiliations:** 1 Division of Gastroenterology and Hepatology, Department of Internal Medicine, School of Medicine, Keio University, 35 Shinanomachi, Shinjuku-Ku, Tokyo, 160-8582 Japan; 2 Department of Clinical Epidemiology and Health Economics, School of Public Health, The University of Tokyo, Tokyo, Japan; 3 CentraCare, Interventional Endoscopy Program, St. Cloud Hospital, St. Cloud, MN USA; 4 Division of Gastroenterology, Hepatology and Nutrition, University of Minnesota, Minneapolis, MN USA

**Keywords:** Ringer’s, Normal saline, Mortality and acute pancreatitis

## Abstract

**Background:**

Fluid resuscitation is fundamental in acute pancreatitis (AP) treatment. However, the optimal choice between normal saline (NS) and Ringer's solution (RS), and its impact on mortality in critically ill patients, remains controversial. This retrospective cohort study, utilizing a national Japanese inpatient database, investigates this question.

**Methods:**

Using the Japanese Diagnosis Procedure Combination database between July 2010 and March 2021, we identified adult patients hospitalized in intensive care units (ICU) or high-dependency care units (HDU) for AP who survived at least three days and received sufficient fluid resuscitation (≥ [10 ml/kg/hr*1 h + 1 ml/kg/hr*71 h] ml) within three days of admission including emergency room infusions. Patients were classified into groups based on the predominant fluid type received: the NS group (> 80% normal saline) and the RS group (> 80% Ringer's solution). Propensity score matching was employed to reduce potential confounding factors and facilitate a balanced comparison of in-hospital mortality between the two groups.

**Results:**

Our analysis included 8710 patients with AP. Of these, 657 (7.5%) received predominantly NS, and 8053 (92.5%) received predominantly RS. Propensity score matching yielded 578 well-balanced pairs for comparison.

The NS group demonstrated significantly higher in-hospital mortality than the RS group (12.8% [474/578] vs. 8.5% [49/578]; risk difference, 4.3%; 95% confidence interval, 0.3% to 8.3%).

**Conclusions:**

In patients admitted to ICU or HDU with AP receiving adequate fluid resuscitation, RS can be a preferred infusion treatment compared to NS.

**Supplementary Information:**

The online version contains supplementary material available at 10.1186/s40560-024-00738-y.

## Introduction

Acute pancreatitis (AP) is a common gastrointestinal disorder with significant healthcare costs exceeding 2.7 billion dollars annually in the United States alone [1]. Hospitalization rates are substantial, and despite an overall mortality of 2%, severe acute pancreatitis (SAP) carries a mortality rate nearing 20% and up to 30% in critically ill patients [2–4].

Early fluid replacement therapy is the cornerstone of AP management according to guidelines, with normal saline (NS) and Ringer's solution (RS) being the most commonly used solutions [5, 6]. While several systematic reviews and meta-analyses suggest potential advantages of RS in reducing local complications, severity, intensive care unit (ICU) admission, and pancreatic necrosis [7–11], its impact on mortality remains less clear. This lack of clarity may be due to the limited sample sizes of included randomized controlled trials (RCTs) or less severely ill patients [12–16].

To address this uncertainty, we conducted a study comparing in-hospital mortality in critically ill patients between AP patients treated with NS or RS, utilizing a large, nationwide Japanese inpatient database.

## Methods

### Study design and dataset

This retrospective cohort study employed a propensity score-matched (PSM) design to compare the clinical efficacy of different fluid resuscitation strategies in AP. We analyzed data from the Diagnosis Procedure Combination (DPC) database, a nationwide Japanese database containing discharge abstracts and administrative claims data from over 1200 acute-care hospitals [17]. This database provides the following patient-level data: age, sex, diagnoses (ICD-10 codes), daily procedures (Japanese medical procedure codes), daily drug administrations, and admission/discharge status. Previous validation studies demonstrate high specificity for diagnoses and procedures within this database [18]. The study was approved by the Institutional Review Board of The University of Tokyo (Approval number 3501-(5); May 19th, 2021).

### Study population

The study period spanned from July 2010 to March 2021. We defined an ICU as a separate unit that provides critical care services, with at least one physician present on-site 24 h a day, round-the-clock nursing care, the necessary equipment to care for critically ill patients, and a nurse-to-patient ratio greater than 1:2. The definition of a high-dependency care unit (HDU) in our study was similar to that of an ICU, with the exception of the required nurse-to-patient ratio, which could be 1:3, 1:4, or 1:5. To identify patients admitted to ICU/HDU, we used specific Japanese medical procedure codes, which are listed in Supplemental Table 1.

**Table 1:** Baseline characteristics before and after propensity score matching

	Before propensity score matching			After propensity score matching		
	Saline *N* = 657	Ringer’s *N* = 8,053	SD	Saline *N* = 578	Ringer’s *N* = 578	SD
Age, years, median (IQR)	66.0 (49.0–77.0)	63.0 (46.0–76.0)	0.119	66.0 (49.0–78.0)	64.0 (49.0–77.0)	0.041
Male, n (%)	445 (67.7)	5195 (64.5)	0.068	388 (67.1)	386 (66.8)	0.007
Smoking history, n (%)						
Nonsmoker	305 (46.4)	3633 (45.1)	0.026	263 (45.5)	252 (43.6)	0.038
Current/past smoker	262 (39.9)	3467 (43.1)	−0.064	235 (40.7)	245 (42.4)	−0.035
Unknown	90 (13.7)	953 (11.8)	0.056	80 (13.8)	81 (14.0)	−0.005
BMI at admission, kg/m^2^, n (%)						
< 18.5	100 (15.2)	1019 (12.7)	0.074	87 (15.1)	87 (15.1)	0
18.5–24.9	365 (55.6)	4842 (60.1)	−0.093	323 (55.9)	339 (58.7)	−0.056
25–29.9	124 (18.9)	1534 (19.0)	−0.004	108 (18.7)	99 (17.1)	0.04
≥ 30.0	43 (6.5)	436 (5.4)	0.048	37 (6.4)	37 (6.4)	0
Missing	25 (3.8)	222 (2.8)	0.059	23 (4.0)	16 (2.8)	0.068
Physical function at admission, n (%)						
Independent (Barthel Index 100)	382 (58.1)	3972 (49.3)	0.178	326 (56.4)	320 (55.4)	0.021
Slight/moderate dependence (Barthel Index 61–99)	28 (4.3)	650 (8.1)	−0.159	26 (4.5)	28 (4.8)	−0.014
Total/severe dependence (Barthel Index 0–60)	112 (17.0)	2032 (25.2)	−0.201	108 (18.7)	117 (20.2)	−0.038
Missing	135 (20.5)	1399 (17.4)	0.081	118 (20.4)	113 (19.6)	0.022
JCS at admission, n (%)						
Clear 0	462 (70.3)	6679 (82.9)	−0.301	423 (73.2)	419 (72.5)	0.017
Confusion 1–3	128 (19.5)	1132 (14.1)	0.146	106 (18.3)	106 (18.3)	0
Somnolence 10–30	35 (5.3)	172 (2.1)	0.169	28 (4.8)	29 (5.0)	−0.009
Coma 100–300	32 (4.9)	70 (0.9)	0.241	21 (3.6)	24 (4.2)	−0.031
Charlson comorbidity index, n (%)						
0	269 (40.9)	4519 (56.1)	−0.307	250 (43.3)	243 (42.0)	0.025
1	193 (29.4)	2287 (28.4)	0.022	181 (31.3)	182 (31.5)	−0.004
≥ 2	195 (29.7)	1247 (15.5)	0.344	147 (25.4)	153 (26.5)	−0.025
Comorbidity of CKD, n (%)	102 (15.5)	128 (1.6)	0.514	56 (9.7)	58 (10.0)	−0.013
Fiscal year at admission, n (%)						
2010–2014	297 (45.2)	2869 (35.6)	0.196	258 (44.6)	264 (45.7)	−0.021
2015–2017	205 (31.2)	2744 (34.1)	−0.061	183 (31.7)	191 (33.0)	−0.03
2018–2020	155 (23.6)	2440 (30.3)	−0.152	137 (23.7)	123 (21.3)	0.055
Admission on a weekend, n (%)	167 (25.4)	2322 (28.8)	−0.077	146 (25.3)	154 (26.6)	−0.031
Ambulance use, n (%)	411 (62.6)	4525 (56.2)	0.13	364 (63.0)	358 (61.9)	0.021
Prognosis factor, n (%)						
0	128 (19.5)	2717 (33.7)	−0.327	123 (21.3)	127 (22.0)	−0.016
1	100 (15.2)	2123 (26.4)	−0.277	95 (16.4)	92 (15.9)	0.013
2	76 (11.6)	1154 (14.3)	−0.082	69 (11.9)	71 (12.3)	−0.01
3	74 (11.3)	602 (7.5)	0.13	68 (11.8)	58 (10.0)	0.059
4	50 (7.6)	378 (4.7)	0.122	44 (7.6)	49 (8.5)	−0.036
5	48 (7.3)	166 (2.1)	0.25	35 (6.1)	40 (6.9)	−0.041
6	28 (4.3)	96 (1.2)	0.189	21 (3.6)	18 (3.1)	0.032
7	14 (2.1)	43 (0.5)	0.14	10 (1.7)	8 (1.4)	0.03
8	8 (1.2)	15 (0.2)	0.124	6 (1.0)	6 (1.0)	0
9	2 (0.3)	12 (0.1)	0.033	1 (0.2)	1 (0.2)	0
Unknown	129 (19.6)	747 (9.3)	0.298	106 (18.3)	108 (18.7)	−0.01
CT grade score, n (%)						
0	135 (20.5)	1,633 (20.3)	0.007	124 (21.5)	138 (23.9)	−0.06
1	88 (13.4)	1,516 (18.8)	−0.148	76 (13.1)	54 (9.3)	0.104
2	171 (26.0)	2,904 (36.1)	−0.218	158 (27.3)	161 (27.9)	−0.011
3	63 (9.6)	656 (8.1)	0.051	56 (9.7)	51 (8.8)	0.03
4	32 (4.9)	251 (3.1)	0.09	26 (4.5)	24 (4.2)	0.018
Unknown	168 (25.6)	1,093 (13.6)	0.306	138 (23.9)	150 (26.0)	−0.053
Ventilator use, n (%)	75 (11.4)	170 (2.1)	0.377	46 (8.0)	45 (7.8)	0.007
Renal replacement therapy, n (%)	154 (23.4)	93 (1.2)	0.721	77 (13.3)	75 (13.0)	0.011
Catecholamine use, n (%)	103 (15.7)	415 (5.2)	0.35	78 (13.5)	71 (12.3)	0.04

*SD* standardized difference, *IQR* interquartile range, *BMI* body mass index, *JCS* Japan Coma Scale, *CKD* chronic renal disease, *CT* computerized tomography

The following exclusion criteria were applied:

1. Lack of emergency admission status.
2. Length of hospital stay ≤ 3 days.
3. Inadequate fluid resuscitation (defined as < 10 ml/kg bolus over one hour followed by < 1 ml/kg/hour over the subsequent 71 h).
4. Mixed fluid resuscitation strategy where neither NS nor RS accounted for at least 80% of the total fluids administered during hospitalization.

### Treatment groups

Patients were classified based on the predominant type of external fluid administered within the first three days of admission:

- NS Group: received NS for ≥ 80% of total fluid volume.
- RS Group: received Ringer's lactate, Ringer's acetate, or Ringer's carbonate for ≥ 80% of total fluid volume.

### Outcomes

- Primary outcome: in-hospital mortality.
- Secondary outcomes:
  - Development of SAP based on the Revised Atlanta Classification [18]. SAP is defined as persistent organ failure in one or more systems (cardiovascular, renal, respiratory).
    ο Persistent cardiovascular failure: requiring vasopressors/inotropes > 3 days.
    ο Persistent renal failure: requiring renal replacement therapy > 3 days.
    ο Persistent respiratory failure: requiring mechanical ventilation > 3 days.
  - Intervention for necrotizing AP (necrosectomy or drainage for symptomatic walled-off necrosis (WON) or acute necrotic collection (ANC) during hospitalization; delayed interventions during re-hospitalization were excluded).
  - Length of hospital stay.
  - Length of ICU/HDU stay.
  - Total hospitalization costs (1 USD ≈ 110 JPY).

### Covariates

We considered the following covariates for their potential impact on outcomes:

**Patient characteristics**

- Age
- Sex
- Smoking history
- Body mass index (BMI) at admission
- Physical function (Barthel Index score at admission [19]
- Level of consciousness (Japan Coma Scale score at admission [20]
- Comorbidities (Charlson Comorbidity Index score and chronic renal disease).

**Admission factors**

- Fiscal year
- Weekend admission (Saturday or Sunday)
- Ambulance use.

**Disease and initial treatment factors**

- AP severity indicators (Japanese Ministry of Health, Labour and Welfare scoring system [21]
- CT grade score (Japanese Ministry of Health, Labour and Welfare scoring system [22]
- Ventilator use on arrival
- Renal replacement therapy on arrival
- Vasopressor/inotrope use on arrival.

### Statistical analysis

PSM was employed to reduce bias and facilitate a balanced comparison of in-hospital mortality between the NS and RS groups [23]. Propensity scores were calculated using a multivariable logistic regression model where the treatment group assignment (NS vs. RS) was the dependent variable, and all covariates listed in Table 1 were independent variables. One-to-one nearest-neighbor matching without replacement was performed using a caliper width of 20% of the standard deviation of propensity scores [23]. Standardized differences were calculated to assess the balance of covariates, with absolute values ≤ 10% indicating negligible imbalance between groups [24]. After PSM, a generalized linear model compared outcomes between groups, accounting for clustering effects within individual hospitals. Differences and 95% confidence intervals (CIs) were calculated using the identity link function, regardless of outcome type.

Several sensitivity analyses were performed to assess the robustness of our findings:

1. Dialysis exclusion: Analysis was repeated excluding patients receiving dialysis within the first day of admission, as their treatment may have favored the potassium-free NS.
2. Inverse probability of treatment weighting (IPTW): IPTW analyses were conducted as an alternative to PSM, using stabilized weighting for greater precision and control over type I error.
3. Fluid type proportion thresholds: Analyses were repeated for populations receiving > 70% and > 90% of their fluid volume as either NS or RS.
4. Total fluid volume stratification: Analyses were performed after dividing patients into low-volume and high-volume fluid resuscitation groups, based on the median total fluid volume within the first three days of admission.

## Results

**Fig. 1:**
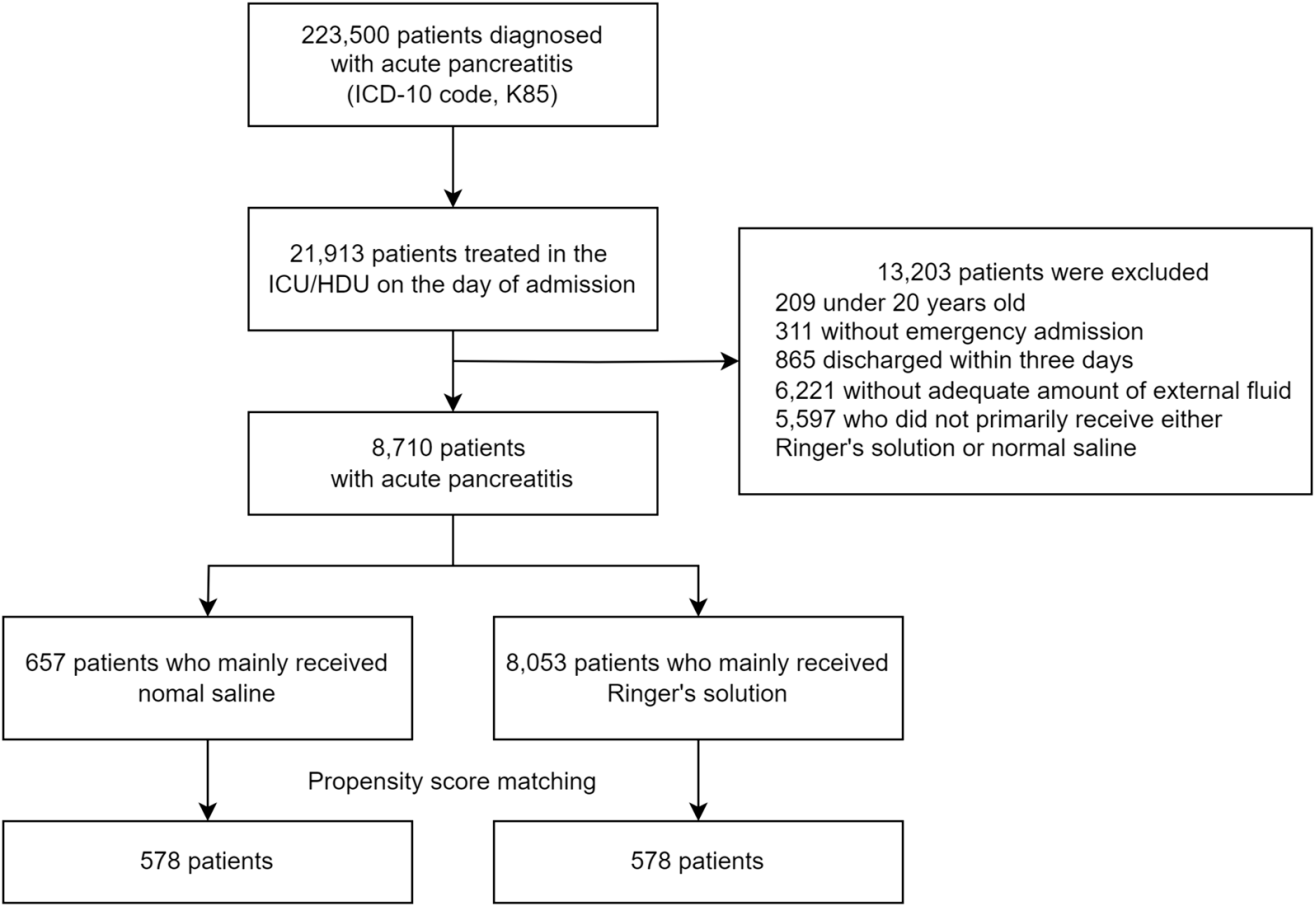
Study flow diagram of patients admitted to intensive care unit or high-dependency care unit

Out of 223,500 patients hospitalized for AP, 8,710 met the inclusion criteria after applying exclusions (Fig. 1). Of these, 657 (7.5%) received predominantly NS, and 8053 (92.5%) received predominantly RS. PSM yielded 578 well-balanced pairs (Table 1). Importantly, no significant differences were found in the total volume of fluid administered within the first three days between the NS and RS groups, both before and after PSM (Table 2).

**Table 2:** Outcomes and treatment before and after propensity score matching

	Before propensity score matching		After propensity score matching		
	Saline *N* = 657	Ringer’s *N* = 8053	Saline *N* = 578	Ringer’s *N* = 578	*P*-value
Primary outcome					
In-hospital mortality	104 (15.8)	275 (3.4)	74 (12.8)	49 (8.5)	0.003
Secondary outcomes					
Severe acute pancreatitis	310 (47.2)	997 (12.4)	232 (40.1)	166 (28.7)	0.008
Persistent organ failure					
Cardiovascular	135 (20.5)	476 (5.9)	101 (17.5)	78 (13.5)	0.096
Renal	237 (36.1)	281 (3.5)	162 (28.0)	101 (17.5)	0.012
Respiratory	167 (25.4)	643 (8.0)	123 (21.3)	110 (19.0)	0.51
Intervention for necrotizing AP	37 (5.6)	205 (2.5)	28 (4.8)	24 (4.2)	0.64
Length of hospital stay	23.0 (14–40)	16.0 (11–25)	23.0 (14–39)	20.0 (13–31)	0.14
Length of ICU/HDU stay	5.0 (2.0–11.0)	3.0 (2.0–6.0)	5.0 (2.0–10.0)	4.0 (2.0–9.0)	0.90
Total hospitalization costs	13,924 (8378–26,650)	8806 (6087–13376)	12,886 (7999–22,839)	11,459 (7265–19,918)	0. 57
Treatment					
Total volume of external fluid administered for three days	8500 (6350–12400)	8150.0 (6450–10500)	8423 (6250–11996)	8800 (6500–12350)	0.60
The volume of NS administered for three days	7900 (6000–11300)	800 (500–1200)	7749 (5950–11050)	950 (600–1500)	< 0.001
The volume of RS administered for three days	500 (0–1000)	7500 (5500–9500)	500 (0–1000)	8000 (6000–11000)	< 0.001

ICU; intensive care unit, HDU; high-dependency care unit, AP; acute pancreatitis, NS; normal saline, RS; Ringer's solution

### Key findings after PSM

**Fig. 2:**
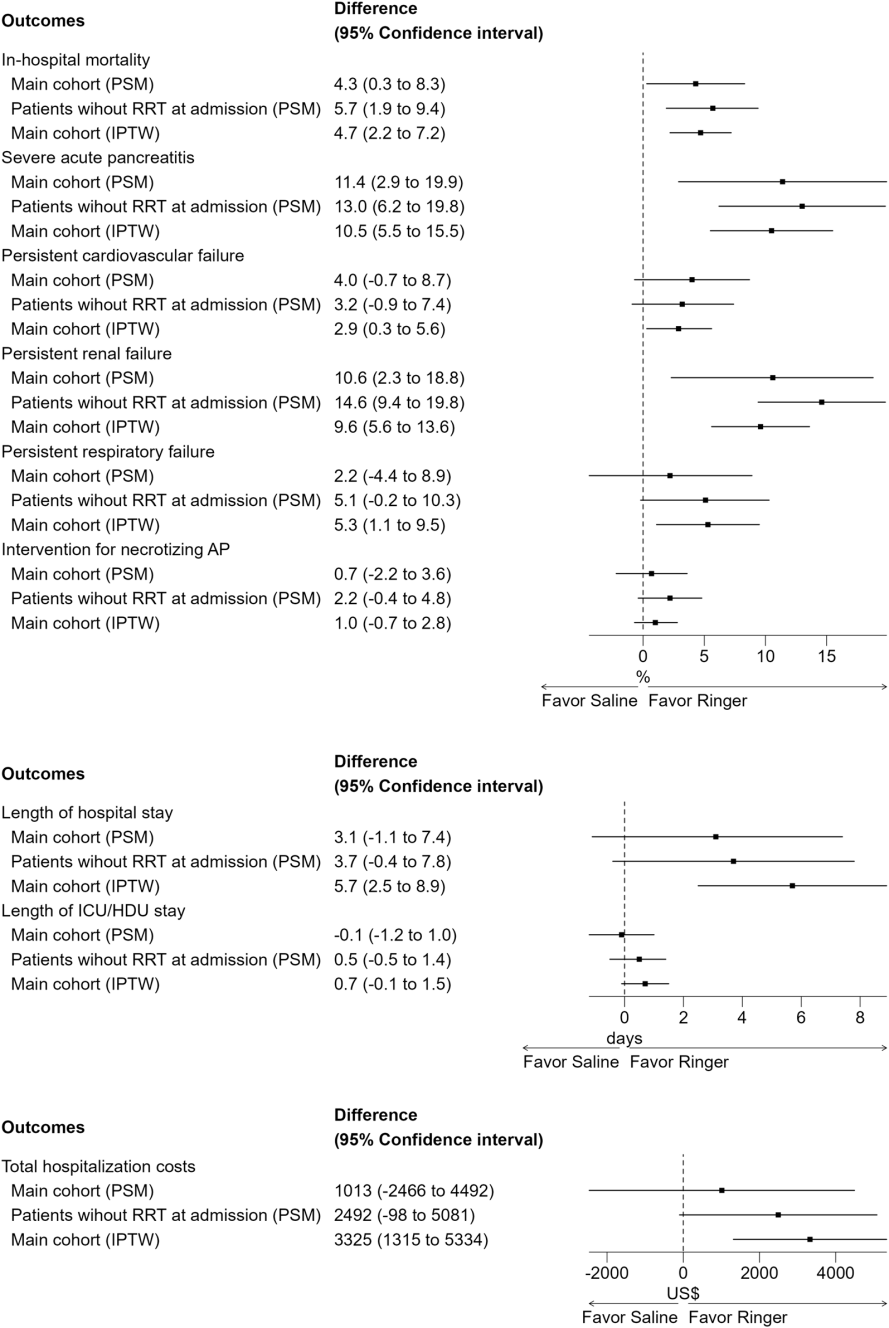
The difference in outcomes between the normal saline and Ringer’ solution groups

- Mortality: The NS group had significantly higher in-hospital mortality than the RS group (12.8% vs. 8.5%; risk difference, 4.3%; 95% CI 0.3–8.3%).
- Secondary outcomes: The NS group also demonstrated significantly worse outcomes in the SAP and persistent renal organ failure (Table 2, Fig. 2). No significant difference was found for persistent organ failure (cardiovascular and respiratory), intervention for necrotizing AP, length of hospital stay, length of ICU/HDU stay, total hospitalization costs.

### Sensitive analysis

#### Dialysis-free patients subgroups

Excluding 247 patients receiving renal replacement therapy on admission, we still observed significantly higher in-hospital mortality in the NS group (11.5% vs. 5.9%; risk difference, 5.6%; 95% CI, 1.9–9.4%). Additional outcomes are presented in Supplementary Table 2 and Figure 2.

#### IPTW analysis

In the IPTW cohort, we found significantly higher in-hospital mortality for the NS group (8.5% vs. 3.8%; risk difference, 4.7%; 95% CI 2.2–7.2%). The NS group also showed significantly worse outcomes across most other endpoints, except intervention for necrotizing AP (Supplementary Table 3 and Figure 2).

#### Different fluid proportion thresholds

Sensitivity analyses using alternate definitions for predominant fluid use (> 70% and > 90% NS or RS) confirmed our main findings. Supplementary Tables 4 and 5 relay detailed results.

#### Fluid volume stratification

Dividing patients by low/high fluid resuscitation volume (median 6400 mL [5500–7300] and 10,600 mL [9250–13500], respectively), we found:

- Low-volume: NS group mortality 9.0% vs. 4.3% for RS (risk difference 4.7%; 95% CI 0.2%–9.2%, *p* = 0.04) after matching 256 patients per group (Supplementary Table 6).
- High-Volume: NS group mortality 15.9% vs. 11.7% for RS (risk difference 4.2%; 95% CI − 1.2–9.7%, *p* = 0.13) after matching 308 patients per group (Supplementary Table 7).

## Discussion

The choice of fluid for treating AP remains a topic of ongoing debate, with previous research yielding mixed results [25, 26]. While RCTs and meta-analyses have not consistently demonstrated a mortality difference between NS and RS [7–16], our large-scale observational study suggests that using RS in adults admitted to ICU/HDU with AP may offer a significant survival benefit. We found a risk difference of 4.3% in favor of RS. Furthermore, our findings associate NS administration with a higher risk of developing SAP and persistent renal organ failure. Various sensitivity analyses also showed that the absolute differences for each outcome (mortality; 4.2–5.6%, SAP; 10.1–14.5%, and persistent renal organ failure; 9.6–14.6%) were in broad agreement.

Earlier RCTs investigating fluid choice in AP may have been underpowered to detect mortality differences due to their small sample sizes and the inclusion of less severely ill patients who may not have required ICU/HDU admission. [12–16]. Similarly, previous meta-analyses suggested improvements in certain outcomes but could not definitively establish a difference in mortality [7–11]. A recent meta-analysis reported lower mortality associated with RS, but only three patients died across the five included studies, and the results might be less reliable for adults due to the inclusion of both adult and pediatric studies [27].

We observed a high overall mortality rate in our study (12.8% with NS and 8.5% with RS) because we focused specifically on critically ill AP patients admitted to the ICU or HDU. Although a RCT would require approximately 1,126 total patients to confirm the 4.3% absolute mortality difference we observed (assuming 90% power and α = 0.05), our large-scale analysis utilizing 578 propensity score-matched pairs from a nationwide adult inpatient database provides compelling evidence that NS is associated with higher mortality compared to RS in the treatment of critically ill ICU/HDU patients with AP. SAP develops in about 20% of AP cases [28, 29], but our subject has a higher percentage of SAP with 40.1% with NS and 28.7% with RS because our analysis focused on critically ill ICU/HDU patients. Although the results of this study may be applicable to a limited number of subjects, the high incidence of AP, with over 280,000 hospitalizations annually in the US alone [1], and the substantial mortality difference suggest a significant clinical impact. Reducing the mortality rate of these severely ill patients could prevent a large number of deaths each year.

RS offers several potential advantages over NS in the management of AP, primarily due to its closer resemblance to the electrolyte and acid–base composition of plasma [30, 31]. RS contains calcium, which may reduce systemic inflammation by binding non-esterified fatty acids [32]. Additionally, the short-chain fatty acids found in RS may modulate inflammatory responses and promote anti-inflammatory pathways [13]. Furthermore, RS may improve renal blood flow and counter chloride-mediated vasoconstriction, potentially offering protection against acute kidney injury compared to NS [32–34]. Our study consistently found RS to be associated with significantly lower persistent renal failure, supporting this mechanism. While NS has advantages in volume expansion and lower cost, these are most relevant in cases of hypovolemic shock. In patients with AP experiencing distributive shock, RS appears to be the superior choice [7–11].

It is important to acknowledge the limitations of our retrospective observational study, primarily the potential for confounding due to the lack of randomization, as fluid choice was at the provider’s discretion and could be influenced by patient characteristics. To address these shortcomings, we employed propensity score matching and several sensitivity analyses, and our consistent results strengthen the reliability of our findings. However, unmeasured confounders, such as catecholamine dosage data and renal failure developed after hospitalization, may persist. Despite these limitations, our findings suggest a potential benefit associated with RS use in AP management. In Japan, while NS use for AP is decreasing, it is still being administered. Given the ease of switching between RS and NS, alongside their cost parity, a shift towards RS could translate to meaningful improvements in patient outcomes on a large scale.

## Conclusion

Adults hospitalized to ICU or HDU for AP who received adequate volumes of NS for fluid resuscitation experienced higher in-hospital mortality compared to those treated with RS. These results align with studies demonstrating RS's potential to reduce complications and improve outcomes. While a large RCT would be ideal to confirm these findings, it may face challenges due to the required sample size and relatively low mortality rates in AP. Considering the observed benefits, ease of implementation, and cost parity between the solutions, RS may be considered the preferred fluid choice in managing adult ICU or HDU patients with AP.

### Supplementary Information

Additional file1  

## Data Availability

The datasets used and analyzed during the current study are available from the corresponding author on reasonable request.

## Abbreviations

AP: Acute pancreatitis
HDU: High-dependency care unit
NS: Normal saline
RS: Ringer's solution
ICU: Intensive care unit
SAP: Severe acute pancreatitis
CI: Confidence interval
