# Supplementary material for: Normal saline versus Ringer’s solution and critical-illness mortality in acute pancreatitis: a nationwide inpatient database study
Source: J Intensive Care. 2024 Jul 15;12:27. doi: 10.1186/s40560-024-00738-y (PMC11247862; doi:10.1186/s40560-024-00738-y)
Supplement: Supplementary file 1 — Additional file1 [file 40560_2024_738_MOESM1_ESM.pdf]

Supplemental Table 1. Codes used to define ICU/HDU using the Japanese medical procedure codes.

Name	Code	Description	Nurse-to-patient ratio
ICU	A3002	Emergency and critical care unit management fee 2	1:2
ICU	A3004	Emergency and critical care unit management fee 4	1:2
ICU	A3011	Intensive care unit management fee 1	1:2
ICU	A3012	Intensive care unit management fee 2	1:2
ICU	A3013	Intensive care unit management fee 3	1:2
ICU	A3014	Intensive care unit management fee 4	1:2
ICU	A301-4	Pediatric intensive care unit management fee	1:2
HDU	A3001	Emergency and critical care unit management fee 1	1:4
HDU	A3003	Emergency and critical care unit management fee 3	1:4
HDU	A301-21	High care unit management fee 1	1:4
HDU	A301-22	High care unit management fee 2	1:5
HDU	A301-3	Stroke care unit management fee	1:3

ICU, intensive care unit; HDU, high-dependency care unit

Supplemental Table 2. Outcomes and treatment of patients without renal replacement therapy at admission before and after propensity score matching

	Before propensity score matching		After propensity score matching		
	Saline N=503	Ringer's N=7,960	Saline N=494	Ringer's N=494	P-value
Primary outcome					
In-hospital mortality	60 (11.9)	255 (3.2)	57 (11.5)	29 (5.9)	0.003
Secondary outcomes					
Severe acute pancreatitis	161 (32.0)	911 (11.4)	154 (31.2)	90 (18.2)	<0.001
Persistent organ failure					
Cardiovascular	67 (13.3)	441 (5.5)	65 (13.2)	49 (9.9)	0.13
Renal	95 (18.9)	196 (2.5)	90 (18.2)	18 (3.6)	<0.001
Respiratory	86 (17.1)	584 (7.3)	82 (16.6)	57 (11.5)	0.062
Intervention for necotizing AP	23 (4.6)	197 (2.5)	23 (4.7)	12 (2.4)	0.093
Length of hospital stay	21.0 (13.0-35.0)	16.0 (11.0-25.0)	21.0 (13.0-34.0)	18.0 (12.0-29.0)	0.074
Length of ICU/HDU stay	4.0 (2.0-8.0)	3.0 (2.0-6.0)	4.0 (2.0-8.0)	4.0 (2.0-8.0)	0.35
Total hospitalization costs	11521.6 (7528.1-18577.4)	8730.9 (6060.0-13177.3)	11200.1 (7501.3-18389.7)	9912.3 (6516.2-15080.2)	0.059
Treatment					
Total volume of external fluid administered for three days	8100.0 (6100.0-11400.0)	8100.0 (6400.0-10500.0)	8150.0 (6200.0-11400.0)	8225.0 (6300.0-10700.0)	0.54
The volume of NS administered for three days	7500.0 (5850.0-10550.0)	800.0 (500.0-1200.0)	7500.0 (5850.0-10550.0)	850.0 (550.0-1300.0)	<0.001
The volume of RS administered for three days	500.0 (0.0-1000.0)	7500.0 (5500.0-9500.0)	500.0 (0.0-1000.0)	7500.0 (5500.0-10000.0)	<0.001

AP; acute pancreatitis, ICU; intensive care unit, HDU; high-dependency care unit, NS; normal saline, RS; Ringer's solution

Supplemental Table 3. Outcomes of patients before and after IPTW

	Before IPTW		After IPTW		
	Saline N=657	Ringer's N=8,053	Saline N=611	Ringer's N=8,099	P-value
Primary outcome					
In-hospital mortality	104 (15.8)	275 (3.4)	52(8.5)	310 (3.8)	<0.001
Secondary outcomes					
Severe acute pancreatitis	310 (47.2)	997 (12.4)	148 (24.2)	1113 (13.8)	<0.001
Persistent organ failure					
Cardiovascular	135 (20.5)	476 (5.9)	58 (9.5)	536 (6.6)	0.032
Renal	237 (36.1)	281 (3.5)	87 (14.2)	374 (4.6)	<0.001
Respiratory	167 (25.4)	643 (8.0)	87 (14.2)	721 (8.9)	0.014
Intervention for necotizing AP	37 (5.6)	205 (2.5)	23 (3.7)	218 (2.7)	0.25
Length of hospital stay	23.0 (14-40)	16.0 (11-25)	21 (13-33)	17 (11-25)	<0.001
Length of ICU/HDU stay	5.0 (2.0-11.0)	3.0 (2.0-6.0)	4.0 (2.0-7.0)	3.0 (2.0-7.0)	0.10
Total hospitalization costs	13924 (8378-26650)	8806 (6087-13376)	10829 (7194-133133)	8933 (6155-13784)	<0.001

IPTW; inverse probability of treatment weighting, AP; acute pancreatitis, ICU; intensive care unit, HDU; high-dependency care unit

Supplemental Table 4. Outcomes of patients receiving normal saline or Ringer's solution accounted for more than 70% of the total volume of external fluid administered for three days

	Before propensity score matching		After propensity score matching			
	Saline N=929	Ringer's N=10,319	Saline N=860	Ringer's N=860	Difference (95% Confidence interval)	P-value
Primary outcome						
In-hospital mortality	145 (15.6)	429 (4.2)	118 (13.7)	80 (9.3)	4.4 (1.1 to 7.7)	0.009
Secondary outcomes						
Severe acute pancreatitis	456 (49.1)	1,606 (15.6)	387 (45.0)	294 (34.2)	10.8 (3.6 to 18.1)	0.003
Persistent organ failure						
Cardiovascular	204 (22.0)	777 (7.5)	167 (19.4)	160 (18.6)	0.8 (-3.2 to 4.8)	0.69
Renal	343 (36.9)	556 (5.4)	277 (32.2)	179 (20.8)	11.4 (4.1 to 18.7)	0.002
Respiratory	250 (26.9)	1,082 (10.5)	215 (25.0)	195 (22.7)	2.3 (-3.3 to 7.9)	0.42
Intervention for necotizing AP	64 (6.9)	295 (2.9)	59 (6.9)	49 (5.7)	1.2 (-1.3 to 3.6)	0.36
Length of hospital stay	24.0 (15.0-42.0)	17.0 (12.0-27.0)	24.0 (15.0-41.0)	21.0 (13.0-34.0)	1.9 (-2.7 to 6.5)	0.43
Length of ICU/HDU stay	5.0 (3.0-11.0)	4.0 (2.0-7.0)	5.0 (2.0-11.0)	5.0 (2.0-11.0)	0.1 (-0.9 to 1.2)	0.80
Total hospitalization costs	14568.0 (8800.7-27481.8)	9390.0 (6384.2-14708.7)	14139.8 (8397.1-25814.1)	12003.9 (7741.8-22024.7)	646 (-2807 to 4100)	0. 71
Treatment						
Total volume of external fluid administered for three days	8800.0 (6600.0-12700.0)	8150.0 (6400.0-10650.0)	8700.0 (6550.0-12350.0)	8900.0 (6600.0-12500.0)		0.44

The volume of NS administered for three days	7600.0 (5828.0-11000.0)	1000.0 (600.0-1600.0)	7500.0 (5774.5-10680.0)	1250.0 (700.0-2125.0)	<0.001
The volume of RS administered for three days	1000.0 (0.0-2000.0)	7000.0 (5500.0-9500.0)	1000.0 (0.0-2000.0)	7500.0 (5500.0-10500.0)	<0.001

AP; acute pancreatitis, ICU; intensive care unit, HDU; high-dependency care unit, NS; normal saline, RS; Ringer's solution

Supplemental Table 5. Outcomes of patients receiving normal saline or Ringer's solution accounted for more than 90% of the total volume of external fluid administered for three days

	Before propensity score matching		After propensity score matching			
	Saline N=442	Ringer's N=3,993	Saline N=357	Ringer's N=357	Difference (95% Confidence interval)	P-value
Primary outcome						
In-hospital mortality	63 (14.3)	94 (2.4)	38 (10.6)	22 (6.2)	4.5 (0.5 to 8.5)	0.028
Secondary outcomes						
Severe acute pancreatitis	197 (44.6)	317 (7.9)	118 (33.1)	73 (20.4)	12.6 (4.4 to 20.1)	0.002
Persistent organ failure						
Cardiovascular	86 (19.5)	141 (3.5)	50 (14.0)	34 (9.5)	4.5 (-0.5 to 9.5)	0.08
Renal	156 (35.3)	80 (2.0)	81 (22.7)	32 (9.0)	13.7 (5.7 to 21.7)	0.001
Respiratory	97 (21.9)	201 (5.0)	58 (16.2)	46 (12.9)	3.4 (-3.1 to 9.8)	0.31
Intervention for necotizing AP	25 (5.7)	70 (1.8)	16 (4.5)	15 (4.2)	0.036 (-0.42 to 1.1)	0.37
Length of hospital stay	23.0 (14.0-39.0)	15.0 (11.0-22.0)	21.0 (14.0-34.0)	17.0 (12.0-28.0)	2.8 (-2.7 to 3.2)	0.85
Length of ICU/HDU stay	5.0 (2.0-10.0)	3.0 (1.0-6.0)	4.0 (2.0-8.0)	4.0 (2.0-7.0)	0.5 (-0.5 to 1.5)	0.34
Total hospitalization costs	13440.9 (8352.6-24496.3)	7840.1 (5529.4-11545.2)	11513.4 (7661.4-18242.0)	9483.6 (6702.8-14193.4)	2506 (-474 to 5485)	0. 099
Treatment						
Total volume of external fluid administered for three days	8250.0 (6200.0-12350.0)	8200.0 (6500.0-10500.0)	8150.0 (6200.0-11850.0)	8200.0 (6400.0-11000.0)		0.067
The volume of NS administered for three days	8150.0 (6000.0-11791.0)	500.0 (200.0-700.0)	8100.0 (6000.0-11300.0)	500.0 (300.0-700.0)		<0.001

The volume of RS administered for three days	0.0 (0.0-500.0)	7500.0 (6000.0-10000.0)	0.0 (0.0-500.0)	8000.0 (6000.0-10500.0)	<0.001

AP; acute pancreatitis, ICU; intensive care unit, HDU; high-dependency care unit, NS; normal saline, RS; Ringer's solution

Supplemental Table 6. Outcomes of patients receiving low volume infusion

	Before propensity score matching		After propensity score matching			
	Saline N=304	Ringer's N=4,038	Saline N=256	Ringer's N=256	Difference (95% Confidence interval)	P-value
Primary outcome						
In-hospital mortality	38 (12.5)	92 (2.3)	23 (9.0)	11 (4.3)	4.7 (0.2 to 9.2)	0.040
Secondary outcomes						
Severe acute pancreatitis	112 (36.8)	252 (6.2)	67 (26.2)	30 (11.7)	14.5 (7.2 to 21.7)	<0.001
Persistent organ failure						
Cardiovascular	39 (12.8)	129 (3.2)	25 (9.8)	11 (4.3)	5.5 (0.9 to 10.0)	0.018
Renal	80 (26.3)	33 (0.8)	38 (14.8)	11 (4.3)	10.5 (4.7 to 16.4)	<0.001
Respiratory	38 (12.5)	85 (2.1)	20 (7.8)	11 (4.3)	3.5 (-0.5 to 7.5)	0.085
Intervention for necotizing AP	9 (3.0)	46 (1.1)	5 (2.0)	5 (2.0)	0 (-2.6 to 2.6)	1.00
Length of hospital stay	22.0 (13.0-35.0)	15.0 (10.0-22.0)	20.5 (13.0-31.5)	17.5 (12.0-30.0)	1.8 (-2.7 to 6.3)	0.42
Length of ICU/HDU stay	4.0 (2.0-8.0)	3.0 (1.0-5.0)	3.0 (2.0-7.0)	3.0 (2.0-7.0)	0.2 (-0.9 to 1.3)	0.71
Total hospitalization costs	11932.9 (7522.3-17959.0)	7701.5 (5501.2-11113.5)	10671.7 (7220.9-16205.3)	9413.5 (6308.4-14272.4)	1669 (-499 to 3836)	0.13
Treatment						
Total volume of external fluid administered for three days	5500 (4675-6200)	5600 (5000-6400)	5500 (4650-6200)	5600 (4900-6250)		0.098
The volume of NS administered for three days	5100 (4300-5950)	500 (100-700)	5100 (4300-5950)	500 (100-700)		<0.001

The volume of RS administered for three days	0 (0-500)	5000 (4500-6000)	0 (0-500)	5000 (4500-5500)	<0.001

AP; acute pancreatitis, ICU; intensive care unit, HDU; high-dependency care unit, NS; normal saline, RS; Ringer's solution

Supplemental Table 7. Outcomes of patients receiving high volume infusion

	Before propensity score matching		After propensity score matching			
	Saline N=353	Ringer's N=4,015	Saline N=308	Ringer's N=308	Difference (95% Confidence interval)	P-value
Primary outcome						
In-hospital mortality	66 (18.7)	183 (4.6)	49 (15.9)	36 (11.7)	4.2 (-1.2 to 9.7)	0.13
Secondary outcomes						
Severe acute pancreatitis	198 (56.1)	745 (18.6)	153 (49.7)	122 (39.6)	10.1 (-0.9 to 21.0)	0.07
Persistent organ failure						
Cardiovascular	96 (27.2)	347 (8.6)	68 (22.1)	67 (21.8)	0.3 -(0.3 to 3.3)	0.09
Renal	157 (44.5)	248 (6.2)	114 (37.0)	75 (24.4)	12.7 (1.3 to 24.0)	0.028
Respiratory	129 (36.5)	558 (13.9)	98 (31.8)	90 (29.2)	2.6 (-0.8 to 12.8)	0.50
Intervention for necotizing AP	28 (7.9)	159 (4.0)	24 (7.8)	17 (5.5)	2.3 (-1.9 to 6.4)	0.28
Length of hospital stay	23.0 (15.0-45.0)	18.0 (13.0-28.0)	23.0 (15.0-44.0)	21.0 (14.0-36.5)	3.4 (-2.9 to 9.7)	0.29
Length of ICU/HDU stay	6.0 (4.0-14.0)	4.0 (2.0-8.0)	6.0 (3.0-13.0)	6.0 (3.0-14.0)	0.2 (-1.2 to 1.6)	0.79
Total hospitalization costs	16522.9 (9256.9-35822.2)	10184.7 (6869.2-16208.9)	15389.8 (9034.2-35255.3)	13220.2 (7973.1-27367.7)	2712 (-1916 to 7340)	0. 25
Treatment						
Total volume of external fluid administered for three days	12000.0 (9800.0-15200.0)	10550.0 (9200.0-12850.0)	11850.0 (9700.0-14950.0)	11586.8 (9500.0-15150.0)		<0.001
The volume of NS administered for three days	11050.0 (9000.0-14300.0)	1100.0 (700.0-1600.0)	10900.0 (8900.0-14225.0)	1367.2 (841.0-1950.0)		<0.001
The volume of RS administered for three days	1000.0 (0.0-1500.0)	9500.0 (8500.0-11500.0)	500.0 (0.0-1500.0)	10000.0 (8500.0-14000.0)		<0.001

AP; acute pancreatitis, ICU; intensive care unit, HDU; high-dependency care unit, NS; normal saline, RS; Ringer's solution
